# How to Navigate in Different Environments and Situations: Lessons From Ants

**DOI:** 10.3389/fpsyg.2018.00841

**Published:** 2018-05-29

**Authors:** Cody A. Freas, Patrick Schultheiss

**Affiliations:** ^1^Department of Biological Sciences, Macquarie University, Sydney, NSW, Australia; ^2^Department of Psychology, University of Alberta, Edmonton, AB, Canada; ^3^Research Center on Animal Cognition, Center for Integrative Biology, French National Center for Scientific Research, Toulouse University, Toulouse, France

**Keywords:** navigation, ants, path integration, sky compass, terrestrial panorama, landmarks, central complex, mushroom bodies

## Abstract

Ants are a globally distributed insect family whose members have adapted to live in a wide range of different environments and ecological niches. Foraging ants everywhere face the recurring challenge of navigating to find food and to bring it back to the nest. More than a century of research has led to the identification of some key navigational strategies, such as compass navigation, path integration, and route following. Ants have been shown to rely on visual, olfactory, and idiothetic cues for navigational guidance. Here, we summarize recent behavioral work, focusing on how these cues are learned and stored as well as how different navigational cues are integrated, often between strategies and even across sensory modalities. Information can also be communicated between different navigational routines. In this way, a shared toolkit of fundamental navigational strategies can lead to substantial flexibility in behavioral outcomes. This allows individual ants to tune their behavioral repertoire to different tasks (e.g., foraging and homing), lifestyles (e.g., diurnal and nocturnal), or environments, depending on the availability and reliability of different guidance cues. We also review recent anatomical and physiological studies in ants and other insects that have started to reveal neural correlates for specific navigational strategies, and which may provide the beginnings of a truly mechanistic understanding of navigation behavior.

## Introduction

Successful navigation requires animals to acquire and apply environmental cues indicating the direction and distance of goal locations. Foraging ants are excellent navigators despite their low visual acuity (Schwarz et al., 2011a; Graham and Philippides, 2017), and their varying navigational strategies have been widely studied (Zeil, 2012; Collett et al., 2013; Cheng et al., 2014; Wehner et al., 2016). These strategies include landmark-based guidance using the panorama (Wehner, 2003; Cheng et al., 2009) and path integration (Collett and Collett, 2000; Wehner, 2003, 2008) with systematic search functioning as a back-up (Schultheiss and Cheng, 2011; Schultheiss et al., 2015). Many of the elements of this navigation toolkit are shared with other social hymenopterans such as bees and wasps, which have been studied in great detail (Collett and Collett, 2002; Cheng, 2006; Zeil, 2012).

Path integration allows the navigator to update its current position relative to the nest by coupling a distance estimate, pedometer-based in ants, with directional estimates from the celestial compass. This coupling results in a working memory-based vector which points the navigator home. As the ant returns to the nest, it runs off this vector which resets once the ant re-enters the nest, yet there is also evidence that ants retain long-term memories of previous vectors (Ziegler and Wehner, 1997).

Landmark use in ants involves the learning of cues present in the panorama (Wehner et al., 1996; Graham and Cheng, 2009; Wystrach et al., 2011). These stored panorama cues are subsequently compared to current views when navigating (Collett et al., 2006; Cheng et al., 2009; Zeil et al., 2014a). How ants acquire, retain, and use both the panorama and other learned cues while foraging continues to be a topic of interest (Knaden and Graham, 2016).

Within this review, we discuss three main avenues of current research in ant navigation. We first summarize the ability of these navigators to learn and retain navigational information from their environment, focusing on panorama cues. Next, we explore current work on how foragers integrate different cues during navigation and how this integration affects cue choice. Finally, we outline the current understanding of the neural architecture underlying these abilities.

## Learning and Memory

### Learning Walks

Using panorama-based navigation first requires the acquisition of cues around the nest through multiple pre-foraging learning walks (Nicholson et al., 1999; Baddeley et al., 2011; Zeil et al., 2014a). During these walks, foragers meander near the nest entrance, likely learning the panorama makeup around the nest (Wehner et al., 2004). Recent work continues to expand our understanding of these walks, focusing on the genus *Cataglyphis*. *Cataglyphis fortis*, a desert species living with few panorama cues, exhibits learning walks that first occur within a few centimeters of the nest entrance, with each subsequent walk becoming wider. These ants typically complete 3–7 walks before the onset of foraging and show clear evidence of improved learning of panorama cues after these learning walks (Fleischmann et al., 2016, 2018). Learning walks appear to be mediated by the environment, as species inhabiting landmark-rich environments (*Cataglyphis aenescens* and *Cataglyphis noda*) will occasionally ‘pirouette’ and turn back to the nest, likely learning panorama cues (Fleischmann et al., 2017). These pirouettes are observed in some barren-habitat species (*Ocymyrmex robustior*, Müller and Wehner, 2010) but not in the widely studied *C. fortis*. Conversely, *C. fortis* foragers walk in loops without stopping, even when landmark cues are artificially present (Fleischmann et al., 2017). Interestingly, the absence of pirouetting does not prevent this species from learning these cues during these walks (Fleischmann et al., 2016). Species-specific differences in terrestrial cue learning during these walks, as well as those of species outside of *Cataglyphis* and *Ocymyrmex* remain largely unstudied and a ripe topic of future research.

### Use of the Panorama

During learned panorama-based navigation, the specific cues in use remain highly debated, as what visual cues and aspects of the panorama are used for directional guidance remains uncertain. Most prevalent models involve view-based matching, where foragers compare stored views with their current view to direct them to goals (Zeil et al., 2003; Möller, 2012). Research has also focused on the use of the skyline pattern/height as navigational cues (Graham and Cheng, 2009). The desert ant *Melophorus bagoti* has been shown to have the ability to use skyline cues through the presence of the UV contrast between the sky and ground to orient successfully as well as retaining skyline cues over long periods (Schultheiss et al., 2016; Freas et al., 2017c). Another view-based strategy of current interest consists of ants’ use of the fractional position of mass of the visual scene when comparing stored views and current views (Lent et al., 2013). Here, ants acquire the fraction of the terrestrial scene to the left and right while facing the goal, comparing these stored views to their current view while navigating. When only a single terrestrial object is visible, foragers appear to learn the position of the object’s center of mass within stored views and attempt to place this center of mass in the same retinal position when navigating (Buehlmann et al., 2016; Woodgate et al., 2016).

### Responding to Panorama Changes

Given that natural cues do not remain constant, ants will occasionally experience changes in the panorama either at their nest or along known routes. Consequently, ants need to be able to respond to these changes while navigating. The nocturnal bull ant *Myrmecia pyriformis* is highly sensitive to panorama changes. When several trees were removed, resulting in small changes to the nest panorama, foragers showed major disruptions in their navigational efficiency, walking slower and less directed. Furthermore, these behavioral changes persisted over multiple nights before returning to pre-change levels, suggesting a period of relearning the new panorama (Narendra and Ramirez-Esquivel, 2017). Yet there appears to be a range of flexibility across species, as recent work in *M. bagoti* suggests foragers learn new panoramas after only one exposure (Freas and Cheng, 2017, 2018a) and can successfully orient to both new and old panoramas for multiple days after a change occurs (Freas et al., 2017c).

Navigating ants also exhibit interesting behaviors when panorama discrepancies occur due to their position in three-dimensional space. When foraging on non-level surfaces, *M. pyriformis* will attempt to roll their head, keeping it close to the horizontal plane. This behavior is believed to reduce visual noise when comparing memorized views with current views, as similarity declines as the view is rotated (Raderschall et al., 2016). An extreme form of this behavior appears to be present while foragers’ bodies are positioned vertically on trees. *Myrmecia midas* foragers perform scans where they roll or pitch their head toward the horizontal plane while the body remains vertical. This behavior may be an attempt to align their current views with memorized views on the ground (Freas et al., 2018).

### Learning Other Cue Sets

While panorama cues are currently the most widely studied form of learning, new research suggests ant navigators can learn a variety of cue sets and associate them with the nest. *Cataglyphis* foragers can also learn associations using local olfactory, magnetic, and vibrational cues. *Cataglyphis noda* will search at locations with locally distinct magnetic, vibrational, and olfactory signatures when these had previously been paired with the nest entrance (Buehlmann et al., 2012a). Additionally, olfactory cues can be learned in association with locations beyond the nest site as part of the foraging route. *Cataglyphis fortis* foragers have been shown to learn odor landmarks along their foraging route after training (Buehlmann et al., 2015). Recent work in the *Cataglyphis* genus is unveiling that the role of olfactory cues has been understudied as a navigational cue set for both nest and food locations (Buehlmann et al., 2012a,b, 2013, 2014, 2015).

## Integration of Navigational Information

### Directional Cue Integration

On featureless saltpans, without visual guidance cues, *C. fortis* foragers use path integration not only to return home, but also to return to previously visited goals. To achieve this, they compare a memorized vector that would lead directly to the goal with the current state of the path integrator (essentially performing vector summation) and deriving a direction in which to move (Collett et al., 1999). This combination of two vector memories, one long-term and one short-term, thus enables them to navigate to a goal. When the previous inbound memory and the current outbound route mismatch consistently, this system adapts by calibrating vectors at recognized sites. Recent experiments on *M. bagoti* revealed that the homeward vector memory recalibrates rapidly, with the inbound vector dominating when the mismatch is small (45°). As the mismatch increases, calibration toward the inbound vector decreases with ants showing no calibration at the maximal mismatch (180°), where the current vector dominates (Freas and Cheng, 2018b).

Such integration of different directional dictates has been found repeatedly in ant navigation studies, and has attracted particular interest as it can show which navigational processes are engaged simultaneously, and how they might be organized in the insect brain (Wehner et al., 2016). Under natural foraging conditions, ants often have multiple sets of guidance cues available simultaneously, and information sharing and integration can occur between different navigational systems. The desert ant *Cataglyphis velox*, for example, navigates home using path integration and memorized terrestrial visual cues. Normally these two systems provide redundant directional information but, when put into conflict, these ants choose intermediate directions. However, during path integration the variance of the directional estimate decreases with vector length, so that after long runs the directional dictate from path integration can be more certain than that from visual memory. The merging of directional information from the two systems has been shown to happen in an optimally weighted manner, taking this relative certainty into account (Wystrach et al., 2015).

### Cue Integration During Learning

The role of learning terrestrial visual cues in such conflict situations has also been explored in more detail in *M. bagoti* (Freas and Cheng, 2017). Foragers restricted to the nest site could not extrapolate visual panorama information to a local (8 m) site. While one exposure to this new panorama was sufficient for successful homing, it did not override a conflicting vector direction. Repeated exposure to the new panorama increased the weighting of these cues, eventually overriding vector information. Interestingly, this pattern of cue choice appears to be dynamic, as terrestrial cues were increasingly discounted with time since last exposure, consistent with the temporal weighting rule (Devenport and Devenport, 1994). View sequence may also be important during landmark learning, as foragers encountering only the inbound view sequence show weaker panorama learning and a higher propensity to switch to vector cues compared to foragers exposed to outbound views (Freas and Cheng, 2018a). Highly visually experienced foragers are not only better at using the panorama for homing, but also better at recognizing changes. Training ants to visit a feeder, Schwarz et al. (2017b) compared visually experienced ants with naïve ants visiting the feeder for the first time. When released in unfamiliar surroundings, naïve ants ran off a longer portion of the path integration vector, while experienced ants broke off their directed travel route earlier. Being familiar with the nest’s surroundings, they could more readily realize that the view was unfamiliar and engage in searching behavior. Buehlmann et al. (2018) investigated the walking speed of *C. fortis* on homeward runs, finding that they slow down when approaching the nest; they are also more alert to visual changes closer to the nest. Interestingly, the relevant cue for these behavioral changes is the completed proportion of the homing vector, suggesting that path integration modulates speed in a way that facilitates the use or learning of visual cues at important locations.

*Myrmecia midas* also orients by both celestial and terrestrial visual cues on outbound trips, and manipulating the direction of polarized overhead light leads to compromises between the directional dictates of celestial and terrestrial cues throughout the outbound journey (Freas et al., 2017b). When orienting on inbound journeys however, they appear to use celestial information only when the accumulated homing vector is large (Freas et al., 2017a). Accordingly, the weighting of celestial cues also scales with vector length (Freas et al., 2017b). Weighted integration of visual cues can therefore be context-dependent. For *C. fortis* ants on salt-pans, the CO_2_-plume emitted by the nest can be an important guidance cue, however, ants will only follow this cue when their homing vector is close to zero (Buehlmann et al., 2012b). This might prevent foragers from mistakenly entering conspecific nests, as CO_2_-plumes are not nest-specific. Such vector-dependence does not apply to food odors, as ants will respond to these regardless of the state of the path integrator (Buehlmann et al., 2013). These findings illustrate that cue integration can function across sensory modalities, in a context-dependent manner.

### Communication Between Navigational Strategies

Information can also be communicated between two navigation systems. Intrigued by the fact that ants can maintain straight compass directions even when walking backward (dragging large food) recent studies on *Cataglyphis* have shown that these backward journeys are frequently interrupted. The ants briefly drop the food and perform small search loops (Pfeffer and Wittlinger, 2016b) or short forward ‘peeks.’ These peeks allow them to use the visual panorama to update their compass heading and transfer this heading to celestial cues (Schwarz et al., 2017a). As the celestial compass can function independently of body orientation, this is then used during backward walking, when the panorama is misaligned (Collett et al., 2017). In other cases, information transfer between systems may not always occur, even when these systems naturally provide redundant information. One such case is odometry, in which *C. fortis* measures the distance traveled by both a stride integrator (Wittlinger et al., 2006) and ventral optic flow (Ronacher and Wehner, 1995). Studying ants that were being carried between sub-colonies, Pfeffer and Wittlinger (2016a) showed that the odometric estimate from optic flow alone was sufficient for subsequent homeward navigation with intact eyes. Ants that were carried the same way, but then had the ventral eye regions covered could not navigate home although their stride integrator was fully functional, showing that odometric information was not communicated between the two systems. Similarly, *M. bagoti* has two parallel systems for perceiving celestial compass cues: through the dorsal rim area of their complex eyes, and through the ocelli on top of their heads. However, after a dog-legged outbound route, only compass information from the eyes is available for path integration, while ocelli information can only be used for reversing the last leg of travel (Schwarz et al., 2011b).

## Neural Mechanisms

To fully comprehend such multi-facetted and flexible navigation behavior on a mechanistic level requires detailed knowledge of the underlying neuroanatomy and physiology. Insects provide the distinct advantage that, though capable of sophisticated behaviors, their central nervous system comprises relatively low neuron numbers (about 1 million in honeybees; Witthöft, 1967), and an understanding should be feasible. Much neurobiological work has focused on the fruit fly *Drosophila* and the honeybee *Apis mellifera*, with considerably less work on ants. Nevertheless, the brains of ants share all the key features with other insects, and with bees in particular (Gronenberg and López-Riquelme, 2004; Bressan et al., 2015).

**FIGURE 1:**
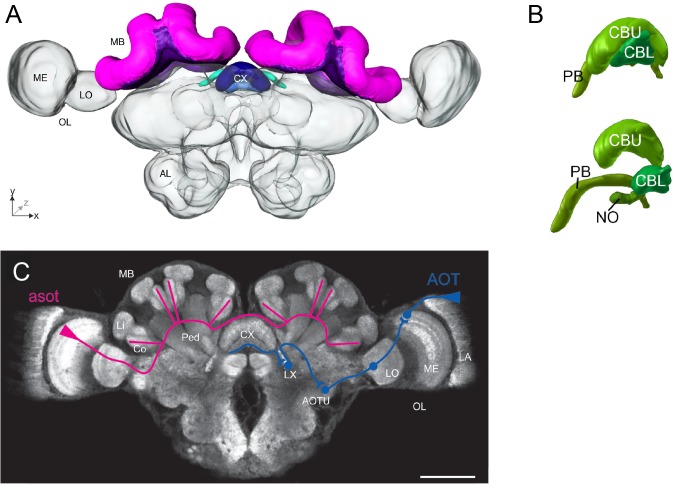
Overview of the main neural structures in the ant brain. **(A)** 3-D reconstruction of an entire *Cataglyphis noda* brain in frontal view, based on confocal laser scanning microscope images. The mushroom bodies (MBs, paired neuropils) are dorsally located (shown in pink and purple), and the central complex (CX, a central neuropil) is located at the midline of the brain (shades of blue). The optic lobes (OLs) with their subcompartments medulla (ME) and lobula (LO) extend laterally toward the compound eyes (not shown), and the antennal lobes (ALs) ventrally toward the antennae (not shown). Adapted from Grob et al. (2017). **(B)** The CX of the ant *Cardiocondyla obscurior*, (above) *in situ* view, (below) exploded view, is subdivided into the central body upper division (CBU), the central body lower division (CBL), the protocerebral bridge (PB), and the noduli (NO). Adapted from Bressan et al. (2015). **(C)** Confocal scan of a *C. noda* brain with anti-synapsin staining in frontal view, showing the different neuropils and schematic representations of the two main visual pathways: the anterior superior optic tract (asot; shown in pink) leads from the ME in the OL to the visual subregions in the collar (Co) of the MB [the Lip (Li) and the peduncle (Ped) are also shown], while the anterior optic tract (AOT, shown in blue) leads from the lamina (LA) in the OL to the CX, via the anterior optic tubercle (AOTU) and the lateral complex (LX); scale bar is 200 μm. Adapted from Grob et al. (2017).

An overview of an ant brain is shown in **Figure 1**. Visual information enters through the optic lobes, while the antennal lobes process olfactory input. The mushroom bodies (MBs) are centers for sensory integration, learning, and memory (Menzel, 2014). The central complex (CX) is involved in memory, visual processing, and sensorimotor processing (Pfeiffer and Homberg, 2014). The neural basis of the sky compass, using polarized light, is currently best understood (Heinze, 2017). Behavioral and physiological findings have revealed that ants perceive the angle of light polarization (POL) through specialized UV-photoreceptors at the dorsal part of the compound eyes (Labhart and Meyer, 1999; Zeil et al., 2014b). A putative neural sky-compass pathway, the anterior optic tract, has been identified (Schmitt et al., 2016), transmitting POL information from the optic lobes to the CX (**Figure 1**). In locusts and *Megalopta* bees, POL angles are anatomically represented in a systematic manner in a subcompartment of the CX, the protocerebral bridge (PB) (Heinze and Homberg, 2007; Stone et al., 2017). In this way, the PB can encode the animal’s global heading, as the direction of POL angles depends on the azimuthal position of the sun. In ants, the neuroanatomy of the CX and its subcompartments is nearly identical and likely functions similarly (Grob et al., 2017).

Recent work in *Drosophila* has shown how the connectivity of the PB and the lower division of the central body (CBL; another CX subcompartment) together form a ring-attractor network, which is able to track changes in heading and update the neural representation accordingly (Seelig and Jayaraman, 2015). Since the CBL also integrates information from POL neurons and speed neurons (Stone et al., 2017), the CX has been successfully modeled as a path integrator (Goldschmidt et al., 2017; Stone et al., 2017). In ants, it remains unclear how speed might be neurally encoded.

The neural mechanisms of other visual navigation strategies, which rely on long-term memories of landscape features, are less well-understood in ants or other insects. It is clear that the MBs play a significant part in visual processing and memory formation (Menzel, 2014), although the CX can be involved in some of these tasks (*Drosophila*: Neuser et al., 2008; ants: Grob et al., 2017). The prominent anterior superior optic tract connects the optic lobes with visual subregions of the MBs (Gronenberg, 2001; **Figure 1**). There is good evidence in ants that these regions are involved in visual memory as they undergo considerable neuroanatomical changes after light exposure (Stieb et al., 2010, 2012). The MBs also contain olfactory subregions that receive neural input from the antennal lobes (Gronenberg and López-Riquelme, 2004). In ants, these subregions go through significant structural changes during the formation of olfactory long-term memories (Falibene et al., 2015) and in bees (*Apis*), the role of MBs in olfactory learning and memory is clearly established (Hourcade et al., 2010). The neural connectivity within *Drosophila* MBs is in fact so well-understood that it has inspired convincing models of their involvement in olfactory learning (Aso et al., 2014); these have since been adapted to model how image-based memories could be stored (Ardin et al., 2016; see also Webb and Wystrach, 2016). It is not yet known how stored visual information might be compared with currently perceived views, or how MB output signals may be converted into motor commands, as prominent neural connections to the CX have not been identified.

To advance our understanding of ant navigation neurobiology in the near future, it remains essential to further elucidate the main circuitry in the ant brain. Neural connections, predicted by our knowledge in related insects and computational models, need to be investigated and verified. Precise neurophysiology on living ants continues to be a key challenge, especially in ecologically relevant contexts. Major advances in *Drosophila* neurobiology have been achieved through neural manipulations on tethered animals, and with recent developments of advanced trackball setups for walking hymenopterans (ants: Dahmen et al., 2017; bees: Schultheiss et al., 2017), such avenues may now be open for ants as well.

## Conclusion

Foraging ants have been key to the study of navigational strategies such as path integration, panorama-based guidance, and the use of a bevy of olfactory, visual, and idiothetic cue sets. This review has focused on three avenues representing the current state of work across multiple species, the learning and storing of navigational cues, the integration of multiple information streams while navigating, and the neural and anatomical structures underlying these strategies. Together, these studies provide the base for forming a mechanistic framework for navigational decision making and behavior.

## Author Contributions

PS conceived the study. CF and PS wrote and revised the manuscript.

## Conflict of Interest Statement

The authors declare that the research was conducted in the absence of any commercial or financial relationships that could be construed as a potential conflict of interest.

## References

[B1] Ardin P. Peng F. Mangan M. Lagogiannis K. Webb B. (2016). Using an insect mushroom body circuit to encode route memory in complex natural environments. *PLoS Comput. Biol.* 12:e1004683. 10.1371/journal.pcbi.1004683 26866692 PMC4750948

[B2] Aso Y. Hattori D. Yu Y. Johnston R. M. Iyer N. A. Ngo T. T. (2014). The neuronal architecture of the mushroom body provides a logic for associative learning. *eLife* 3:e04577. 10.7554/eLife.04577 25535793 PMC4273437

[B3] Baddeley B. Graham P. Philippides A. Husbands P. (2011). Holistic visual encoding of ant-like routes: navigation without waypoints. *Adapt. Behav.* 19 3–15. 10.1177/1059712310395410

[B4] Bressan J. M. Benz M. Oettler J. Heinze J. Hartenstein V. Sprecher S. G. (2015). A map of brain neuropils and fiber systems in the ant *Cardiocondyla obscurior*. *Front. Neuroanat.* 8:166. 10.3389/fnana.2014.00166 25698935 PMC4316776

[B5] Buehlmann C. Fernandes A. S. D. Graham P. (2018). The interaction of path integration and terrestrial visual cues in navigating desert ants: what can we learn from path characteristics? *J. Exp. Biol.* 221:jeb167304. 10.1242/jeb.167304 29146769

[B6] Buehlmann C. Graham P. Hansson B. S. Knaden M. (2014). Desert ants locate food by combining high sensitivity to food odors with extensive crosswind runs. *Curr. Biol.* 24 960–964. 10.1016/j.cub.2014.02.056 24726153

[B7] Buehlmann C. Graham P. Hansson B. S. Knaden M. (2015). Desert ants use olfactory scenes for navigation. *Anim. Behav.* 106 99–105. 10.1016/j.anbehav.2015.04.029

[B8] Buehlmann C. Hansson B. S. Knaden M. (2012a). Desert ants learn vibration and magnetic landmarks. *PLoS One* 7:e33117. 10.1371/journal.pone.0033117 22412989 PMC3296771

[B9] Buehlmann C. Hansson B. S. Knaden M. (2012b). Path integration controls nest-plume following in desert ants. *Curr. Biol.* 22 645–649. 10.1016/j.cub.2012.02.029 22405868

[B10] Buehlmann C. Hansson B. S. Knaden M. (2013). Flexible weighing of olfactory and vector information in the desert ant *Cataglyphis fortis*. *Biol. Lett.* 9:20130070. 10.1098/rsbl.2013.0070 23594568 PMC3645040

[B11] Buehlmann C. Woodgate J. L. Collett T. S. (2016). On the encoding of panoramic visual scenes in navigating wood ants. *Curr. Biol.* 26 2022–2027. 10.1016/j.cub.2016.06.005 27476601

[B12] Cheng K. (2006). “Arthropod navigation: ants, bees, crabs, spiders finding their way,” in *Comparative Cognition: Experimental Explorations of Animal Intelligence*, eds Wasserman E. A. Zentall T. R. (Oxford: Oxford University Press), 189–209.

[B13] Cheng K. Narendra A. Sommer S. Wehner R. (2009). Traveling in clutter: navigation in the Central Australian desert ant *Melophorus bagoti*. *Behav. Processes* 80 261–268. 10.1016/j.beproc.2008.10.015 19049857

[B14] Cheng K. Schultheiss P. Schwarz S. Wystrach A. Wehner R. (2014). Beginnings of a synthetic approach to desert ant navigation. *Behav. Processes* 102 51–61. 10.1016/j.beproc.2013.10.001 24129029

[B15] Collett M. Chittka L. Collett T. S. (2013). Spatial memory in insect navigation. *Curr. Biol.* 23 R789–R800. 10.1016/j.cub.2013.07.020 24028962

[B16] Collett M. Collett T. S. Wehner R. (1999). Calibration of vector navigation in desert ants. *Curr. Biol.* 9 1031–1034. 10.1016/S0960-9822(99)80451-5 10508615

[B17] Collett M. Graham P. Collett T. S. (2017). Insect navigation: what backward walking reveals about the control of movement. *Curr. Biol.* 27 R141–R144. 10.1016/j.cub.2016.12.037 28222290

[B18] Collett T. S. Collett M. (2000). Path integration in insects. *Curr. Opin. Neurobiol.* 10 757–762. 10.1016/S0959-4388(00)00150-1 11240286

[B19] Collett T. S. Collett M. (2002). Memory use in insect visual navigation. *Nat. Rev. Neurosci.* 3 542–552. 10.1038/nrn872 12094210

[B20] Collett T. S. Graham P. Harris R. A. Hempel-de-Ibarra N. (2006). Navigational memories in ants and bees: memory retrieval when selecting and following routes. *Adv. Study Behav.* 36 123–172. 10.1016/S0065-3454(06)36003-2

[B21] Dahmen H. Wahl V. L. Pfeffer S. E. Mallot H. A. Wittlinger M. (2017). Naturalistic path integration of *Cataglyphis* desert ants on an air-cushioned lightweight spherical treadmill. *J. Exp. Biol.* 220 634–644. 10.1242/jeb.148213 28202651

[B22] Devenport L. D. Devenport J. A. (1994). Time-dependent averaging of foraging information in least chipmunks and golden-mantled ground squirrels. *Anim. Behav.* 47 787–802. 10.1006/anbe.1994.1111

[B23] Falibene A. Roces F. Rössler W. (2015). Long-term avoidance memory formation is associated with a transient increase in mushroom body synaptic complexes in leaf-cutting ants. *Front. Behav. Neurosci.* 9:84. 10.3389/fnbeh.2015.00084 25904854 PMC4389540

[B24] Fleischmann P. N. Christian M. Müller V. L. Rössler W. Wehner R. (2016). Ontogeny of learning walks and the acquisition of landmark information in desert ants, *Cataglyphis fortis*. *J. Exp. Biol.* 219 3137–3145. 10.1242/jeb.140459 27481270

[B25] Fleischmann P. N. Grob R. Wehner R. Rössler W. (2017). Species-specific differences in the fine structure of learning walk elements in *Cataglyphis* ants. *J. Exp. Biol.* 220 2426–2435. 10.1242/jeb.158147 28679795

[B26] Fleischmann P. N. Rössler W. Wehner R. (2018). Early foraging life: spatial and temporal aspects of landmark learning in the ant *Cataglyphis noda*. *J. Comp. Physiol. A Neuroethol. Sens. Neural Behav. Physiol.* 10.1007/s00359-018-1260-6 [Epub ahead of print]. 29679143 PMC5966506

[B27] Freas C. A. Cheng K. (2017). Learning and time-dependent cue choice in the desert ant, *Melophorus bagoti*. *Ethology* 123 503–515. 10.1111/eth.12626

[B28] Freas C. A. Cheng K. (2018a). Landmark learning, cue conflict and outbound view sequence in navigating desert ants. *J. Exp. Psychol. Anim. Learn. Cogn.* 10.1037/xan0000178 29975078

[B29] Freas C. A. Cheng K. (2018b). Limits of vector calibration in the Australian desert ant, *Melophorus bagoti*. *Insect. Soc.* 65 141–152. 10.1007/s00040-017-0595-2

[B30] Freas C. A. Narendra A. Cheng K. (2017a). Compass cues used by a nocturnal bull ant, *Myrmecia midas*. *J. Exp. Biol.* 220 1578–1585. 10.1242/jeb.152967 28183865

[B31] Freas C. A. Narendra A. Lemesle C. Cheng K. (2017b). Polarized light use in the nocturnal bull ant, *Myrmecia midas*. *R. Soc. Open Sci.* 4:170598. 10.1098/rsos.170598 28879002 PMC5579118

[B32] Freas C. A. Whyte C. Cheng K. (2017c). Skyline retention and retroactive interference in the navigating Australian desert ant, *Melophorus bagoti*. *J. Comp. Physiol. A.* 203 353–367. 10.1007/s00359-017-1174-8 28447200

[B33] Freas C. A. Wystrach A. Narendra A. Cheng K. (2018). The view from the trees: nocturnal bull ants, *Myrmecia midas*, use the surrounding panorama while descending from trees. *Front. Psychol.* 9:16. 10.3389/fpsyg.2018.00016 29422880 PMC5788958

[B34] Goldschmidt D. Manoonpong P. Dasgupta S. (2017). A neurocomputational model of goal-directed navigation in insect-inspired artificial agents. *Front. Neurorobot.* 11:20. 10.3389/fnbot.2017.00020 28446872 PMC5388780

[B35] Graham P. Cheng K. (2009). Ants use the panoramic skyline as a visual cue during navigation. *Curr. Biol.* 19 R935–R937. 10.1016/j.cub.2009.08.015 19889365

[B36] Graham P. Philippides A. (2017). Vision for navigation: What can we learn from ants? *Arthropod Struct. Dev.* 46 718–722. 10.1016/j.asd.2017.07.001 28751148

[B37] Grob R. Fleischmann P. N. Grübel K. Wehner R. Rössler W. (2017). The role of celestial compass information in *Cataglyphis* ants during learning walks and for neuroplasticity in the central complex and mushroom bodies. *Front. Behav. Neurosci.* 11:226. 10.3389/fnbeh.2017.00226 29184487 PMC5694495

[B38] Gronenberg W. (2001). Subdivisions of hymenopteran mushroom body calyces by their afferent supply. *J. Comp. Neurol.* 436 474–489. 10.1002/cne.1045 11406827

[B39] Gronenberg W. López-Riquelme G. O. (2004). Multisensory convergence in the mushroom bodies of ants and bees. *Acta Biol. Hung.* 55 31–37. 10.1556/ABiol.55.2004.1-4.5 15270216

[B40] Heinze S. (2017). Unraveling the neural basis of insect navigation. *Curr. Opin. Insect Sci.* 24 58–67. 10.1016/j.cois.2017.09.001 29208224 PMC6186168

[B41] Heinze S. Homberg U. (2007). Maplike representation of celestial e-vector orientations in the brain of an insect. *Science* 315 995–997. 10.1126/science.1135531 17303756

[B42] Hourcade B. Muenz T. S. Sandoz J.-C. Rössler W. Devaud J.-M. (2010). Long-term memory leads to synaptic reorganization in the mushroom bodies: a memory trace in the insect brain? *J. Neurosci.* 30 6461–6465. 10.1523/JNEUROSCI.0841-10.2010 20445072 PMC6632731

[B43] Knaden M. Graham P. (2016). The sensory ecology of ant navigation: from natural environments to neural mechanisms. *Annu. Rev. Entomol.* 61 63–76. 10.1146/annurev-ento-010715-023703 26527301

[B44] Labhart T. Meyer E. P. (1999). Detectors for polarized skylight in insects: a survey of ommatidial specializations in the dorsal rim area of the compound eye. *Microsc. Res. Tech.* 47 368–379. 10.1002/(SICI)1097-0029(19991215)47:6<368::AID-JEMT2>3.0.CO;2-Q 10607378

[B45] Lent D. D. Graham P. Collett T. S. (2013). Visual scene perception in navigating wood ants. *Curr. Biol.* 23 684–690. 10.1016/j.cub.2013.03.016 23583550

[B46] Menzel R. (2014). The insect mushroom body, an experience-dependent recoding device. *J. Physiol. Paris* 108 84–95. 10.1016/j.jphysparis.2014.07.004 25092259

[B47] Möller R. (2012). A model of ant navigation based on visual prediction. *J. Theor. Biol.* 305 118–130. 10.1016/j.jtbi.2012.04.022 22554981

[B48] Müller M. Wehner R. (2010). Path integration provides a scaffold for landmark learning in desert ants. *Curr. Biol.* 20 1368–1371. 10.1016/j.cub.2010.06.035 20619653

[B49] Narendra A. Ramirez-Esquivel F. (2017). Subtle changes in the landmark panorama disrupt visual navigation in a nocturnal bull ant. *Philos. Trans. R. Soc. Lond. B Biol. Sci.* 372:20160068. 10.1098/rstb.2016.0068 28193813 PMC5312018

[B50] Neuser K. Triphan T. Mronz M. Poeck B. Strauss R. (2008). Analysis of a spatial orientation memory in *Drosophila*. *Nature* 453 1244–1247. 10.1038/nature07003 18509336

[B51] Nicholson D. J. Judd S. P. D. Cartwright B. A. Collett T. S. (1999). Learning walks and landmark guidance in wood ants (*Formica rufa*). *J. Exp. Biol.* 202 1831–1838. 10359685 10.1242/jeb.202.13.1831

[B52] Pfeffer S. E. Wittlinger M. (2016a). Optic flow odometry operates independently of stride integration in carried ants. *Science* 353 1155–1157. 10.1126/science.aaf9754 27609893

[B53] Pfeffer S. E. Wittlinger M. (2016b). How to find home backwards? Navigation during rearward homing of *Cataglyphis fortis* desert ants. *J. Exp. Biol.* 219 2119–2126. 10.1242/jeb.137786 27445399

[B54] Pfeiffer K. Homberg U. (2014). Organization and functional roles of the central complex in the insect brain. *Annu. Rev. Entomol.* 59 165–184. 10.1146/annurev-ento-011613-162031 24160424

[B55] Raderschall C. A. Narendra A. Zeil J. (2016). Head roll stabilisation in the nocturnal bull ant *Myrmecia pyriformis*: implications for visual navigation. *J. Exp. Biol.* 219 1449–1457. 10.1242/jeb.134049 26994172

[B56] Ronacher B. Wehner R. (1995). Desert ants *Cataglyphis fortis* use self-induced optic flow to measure distances travelled. *J. Comp. Physiol. A* 177 21–27. 10.1007/BF00243395 10708632

[B57] Schmitt F. Stieb S. M. Wehner R. Rössler W. (2016). Experience-related reorganization of giant synapses in the lateral complex: potential role in plasticity of the sky-compass pathway in the desert ant *Cataglyphis fortis*. *Dev. Neurobiol.* 76 390–404. 10.1002/dneu.22322 26138802

[B58] Schultheiss P. Buatois A. Avarguès-Weber A. Giurfa M. (2017). Using virtual reality to study visual performances of honeybees. *Curr. Opin. Insect Sci.* 24 43–50. 10.1016/j.cois.2017.08.003 29208222

[B59] Schultheiss P. Cheng K. (2011). Finding the nest: inbound searching behaviour in the Australian desert ant, *Melophorus bagoti*. *Anim. Behav.* 81 1031–1038. 10.1016/j.anbehav.2011.02.008

[B60] Schultheiss P. Cheng K. Reynolds A. M. (2015). Searching behavior in social Hymenoptera. *Learn. Motiv.* 50 59–67. 10.1016/j.lmot.2014.11.002

[B61] Schultheiss P. Wystrach A. Schwarz S. Tack A. Delor J. Nooten S. S. (2016). Crucial role of ultraviolet light for desert ants in determining direction from the terrestrial panorama. *Anim. Behav.* 115 19–28. 10.1016/j.anbehav.2016.02.027

[B62] Schwarz S. Mangan M. Zeil J. Webb B. Wystrach A. (2017a). How ants use vision when homing backward. *Curr. Biol.* 27 401–407. 10.1016/j.cub.2016.12.019 28111152

[B63] Schwarz S. Wystrach A. Cheng K. (2017b). Ants’ navigation in an unfamiliar environment is influenced by their experience of a familiar route. *Sci. Rep.* 7:14161. 10.1038/s41598-017-14036-1 29074991 PMC5658437

[B64] Schwarz S. Narendra A. Zeil J. (2011a). The properties of the visual system in the Australian desert ant *Melophorus bagoti*. *Arthropod Struct. Dev.* 40 128–134. 10.1016/j.asd.2010.10.003 21044895

[B65] Schwarz S. Wystrach A. Cheng K. (2011b). A new navigational mechanism mediated by ant ocelli. *Biol. Lett.* 7 856–858. 10.1098/rsbl.2011.0489 21733873 PMC3210681

[B66] Seelig J. D. Jayaraman V. (2015). Neural dynamics for landmark orientation and angular path integration. *Nature* 521 186–191. 10.1038/nature14446 25971509 PMC4704792

[B67] Stieb S. M. Hellwig A. Wehner R. Rössler W. (2012). Visual experience affects both behavioral and neuronal aspects in the individual life history of the desert ant *Cataglyphis fortis*. *Dev. Neurobiol.* 72 729–742. 10.1002/dneu.20982 21954136

[B68] Stieb S. M. Muenz T. S. Wehner R. Rössler W. (2010). Visual experience and age affect synaptic organization in the mushroom bodies of the desert ant *Cataglyphis fortis*. *Dev. Neurobiol.* 70 408–423. 10.1002/dneu.20785 20131320

[B69] Stone T. Webb B. Adden A. Weddig N. B. Honkanen A. Templin R. (2017). An anatomically constrained model for path integration in the bee brain. *Curr. Biol.* 27 3069–3085. 10.1016/j.cub.2017.08.052 28988858 PMC6196076

[B70] Webb B. Wystrach A. (2016). Neural mechanisms of insect navigation. *Curr. Opin. Insect Sci.* 15 27–39. 10.1016/j.cois.2016.02.011 27436729

[B71] Wehner R. (2003). Desert ant navigation: how miniature brains solve complex tasks. *J. Comp. Physiol. A* 189 579–588. 10.1007/s00359-003-0431-1 12879352

[B72] Wehner R. (2008). The architecture of the desert ant’s navigational toolkit (Hymenoptera, Formicidae). *Myrmecol. News.* 12 85–96.

[B73] Wehner R. Hoinville T. Cruse H. Cheng K. (2016). Steering intermediate courses: desert ants combine information from various navigational routines. *J. Comp. Physiol. A* 202 459–472. 10.1007/s00359-016-1094-z 27259296

[B74] Wehner R. Meier C. Zollikofer C. (2004). The ontogeny of foraging behaviour in desert ants, *Cataglyphis bicolor*. *Ecol. Entomol.* 29 240–250. 10.1111/j.0307-6946.2004.00591.x

[B75] Wehner R. Michel B. Antonsen P. (1996). Visual navigation in insects: coupling of egocentric and geocentric information. *J. Exp. Biol.* 199 129–140. 9317483 10.1242/jeb.199.1.129

[B76] Witthöft W. (1967). Absolute Anzahl und Verteilung der Zellen im Hirn der Honigbiene. *Z. Morph. Tiere* 61 160–184. 10.1007/BF00298776

[B77] Wittlinger M. Wehner R. Wolf H. (2006). The ant odometer: stepping on stilts and stumps. *Science* 312 1965–1967. 10.1126/science.1126912 16809544

[B78] Woodgate J. L. Buehlmann C. Collett T. S. (2016). When navigating wood ants use the centre of mass of a shape to extract directional information from a panoramic skyline. *J. Exp. Biol.* 219 1689–1696. 10.1242/jeb.136697 26994187

[B79] Wystrach A. Beugnon G. Cheng K. (2011). Landmarks or panoramas: what do navigating ants attend to for guidance? *Front. Zool.* 8:21. 10.1186/1742-9994-8-21 21871114 PMC3177867

[B80] Wystrach A. Mangan M. Webb B. (2015). Optimal cue integration in ants. *Proc. Biol. Sci.* 282:20151484. 10.1098/rspb.2015.1484 26400741 PMC4614770

[B81] Zeil J. (2012). Visual homing: an insect perspective. *Curr. Opin. Neurobiol.* 22 285–293. 10.1016/j.conb.2011.12.008 22221863

[B82] Zeil J. Hofmann M. I. Chahl J. S. (2003). Catchment areas of panoramic snapshots in outdoor scenes. *J. Opt. Soc. Am. A* 20 450–469. 10.1364/JOSAA.20.000450 12630831

[B83] Zeil J. Narendra A. Stürzl W. (2014a). Looking and homing: how displaced ants decide where to go. *Philos. Trans. R. Soc. Lond. B Biol. Sci.* 369 20130034. 10.1098/rstb.2013.0034 24395961 PMC3886322

[B84] Zeil J. Ribi W. A. Narendra A. (2014b). “Polarisation vision in ants, bees and wasps,” in *Polarized Light and Polarization Vision in Animal Sciences*, ed. Hórvath G. (Berlin: Springer), 41–60.

[B85] Ziegler P. E. Wehner R. (1997). Time-courses of memory decay in vector-based and landmark-based systems of navigation in desert ants, *Cataglyphis fortis*. *J. Comp. Physiol. A* 181 13–20. 10.1007/s003590050088

